# Serum biomarker panels for the diagnosis of gastric cancer

**DOI:** 10.1002/cam4.2055

**Published:** 2019-03-14

**Authors:** Dan Wu, Pinglu Zhang, Ji Ma, Jinbo Xu, Li Yang, Weidan Xu, Haifeng Que, Meifen Chen, Hongtao Xu

**Affiliations:** ^1^ Department of Gastrointestinal Surgery Lishui Municipal Central Hospital Lishui Zhejiang China

**Keywords:** antibody array, biomarkers, gastric cancer, serum

## Abstract

Gastric cancer is a leading cause of mortality due to neoplastic disease. Although early detection of gastric cancers can decrease the mortality rate, it remains a diagnostic challenge because of the lack of effective biomarkers. In this study, fifteen gastric cancer patients and ten healthy subjects were recruited to assess novel serum biomarkers for gastric cancer using antibody microarray technology. ELISA was utilized to validate the antibody array results. As a result, compared to the controls, eleven cytokines were found to be significantly increased in gastric cancer, including interferon gamma receptor 1 (IFNGR1), neurogenic locus notch homolog protein 3 (Notch‐3), tumor necrosis factor receptor superfamily member 19L (TNFRSF19L), growth hormone receptor (GHR), signaling lymphocytic activation molecule family 8 (SLAMF8), folate receptor beta (FR‐beta), integrin alpha 5, galectin‐8, erythropoietin‐producing hepatocellular A1 (EphA1), epiregulin, and fibroblast growth factor 12 (FGF‐12) with *P* < 0.05. ELISA validation supported the results of the antibody array. More importantly, most of these eleven cytokines, including IFNGR1, TNFRSF19L, GHR, SLAMF8, FR‐beta, and integrin alpha 5 were discovered to be elevated in gastric cancer serum samples for the first time in this study, suggesting that these proteins may serve as novel biomarkers for the early diagnosis and prognosis determination of gastric cancer.

## 1 INTRODUCTION

Gastric cancer is one of the most common human cancer types, poses a major public health problem globally and is particularly prevalent in Asian populations.^1^ Symptomatology of gastric cancer is rare and nonspecific in the early stages of the disease.^2^ Although surgical resection is an effective therapeutic procedure for gastric cancer and recent advances in chemotherapy have improved the progression‐free survival and overall survival rates, patient prognosis remains poor and the 5‐year survival rate is only about 20% for patients with late stage gastric cancer. Therefore, an early and precise diagnosis is beneficial and critical for ensuring early and effective treatment and for improving the survival rate of gastric cancer patients.

The primary method of diagnosis for gastric cancer is endoscopy and biopsy. However, this procedure relies on the skill of the operator, is invasive and expensive, and causes the patients discomfort and anxiety. Although there have been some advancements in the development of molecular biomarkers for the early detection of gastric cancer in recent years,^3^ useful diagnostic biomarkers for early diagnosis of gastric cancer remain limited. Conventional serum tumor biomarkers, such as carcinoembryonic antigen (CEA), carbohydrate antigen 19‐9 (CA19‐9), carbohydrate antigen 72‐4 (CA72‐4), and carbohydrate antigen 125 (CA125) have been reported to be useful for the early diagnosis, prognostic determination and monitoring of recurrence in gastric cancers.^4^, ^5^ However, most of these serum‐based biomarkers are not recommended for gastric cancer detection due to the limit of specificity and sensitivity in the early stages of gastric cancer.^6^ Thus, it is essential to identify additional biomarkers for effective early diagnosis of gastric cancer.

There are proteomic alterations in the development and progression of diseases. Antibody microarrays are a novel technology rapidly detecting multiple proteins in parallel, and have been applied in many cancers, such as breast cancer and bladder cancer, for biomarker screening.^7^, ^8^ However, no reports regarding the search for gastric cancer serum biomarkers using antibody microarrays have been found. In this study, we aimed to identify serum biomarkers for the improvement of early diagnoses and prognoses of gastric cancer using the antibody microarray technique.

## 2 MATERIALS AND METHODS

### 2.1 Patients

In this study, 15 individuals diagnosed with stage IA (T1N0M0) gastric cancer, based on biopsy specimen analysis and according to the Tumor Node Metastasis (TNM) classification, and who were hospitalized in the Gastrointestinal Surgery Department of Lishui Central Hospital (Lishui, China) in 2017 were recruited. The patients had not yet received chemotherapy or radiotherapy. 10 individuals were recruited as control subjects. The clinical information of the study participants is shown in Table 1. Between the two groups, the age and sex of the subjects were not significantly different. All participants signed informed consent forms prior to their inclusion in this study. Study approval was obtained from the Ethics Committee of the Lishui Central Hospital. Peripheral blood samples were collected from all participants and the serum was separated, frozen, and stored at −80°C according to standard laboratory protocols.

**Table 1:** Clinical data of patients and controls for antibody array detection

Patient	
n	15
Age (mean ± SD), y	58.67 ± 5.84
Sex	46.67% male, 53.33% female
Tumor location	Tunica mucosa
Lymphatic metastasis	no
Distant metastasis	no
Tumor diameter	<3 cm
Histological type	Undifferentiated
Stage	IA
Treatment	no
Control	
n	10
Age (mean ± SD), year	58.10 ± 6.89
Sex	50% male, 50% female
*P*‐value (age, patient vs control)	0.833

### 2.2 Antibody array assay

A human cytokine antibody array (RayBio Human Cytokine Antibody Array Glass series 11, Cat#: GSH‐CYT‐11, RayBiotech, Norcross, GA) that simultaneously detects 40 cytokines was utilized. Briefly, serum samples were incubated with 40 primary antibodies in the arrays overnight at 4°C. The next day, the arrays were washed, a biotin‐conjugated detection antibody mix was added to the array pools and the arrays were incubated for 2 hours at room temperature. The slides were washed again, and Cy3‐conjugated streptavidin was used to bind biotin‐conjugated detection antibodies for a further 2 hours at room temperature. The slides were scanned with an InnoScan 300 Microarray Scanner (Innopsys, France). The signal values were read using Mapix software and were normalized using internal positive controls in the array using the RayBiotech analysis tool, which was specifically designed for the Human Cytokine Antibody Array Glass series 11. This experiment was performed once.

### 2.3 ELISA validation

In order to validate the antibody array results, serum samples from 20 control subjects and 20 gastric cancer patients (information shown in Table 2) were analyzed by ELISA for six cytokines (RayBiotech, Norcross GA, USA. Cat#: ELH‐IFNgR1‐1, ELH‐NOTCH3‐1, ELH‐GHR‐1, ELH‐RELT‐1, ELH‐FOLR2‐1 and ELH‐BLAME‐1) at a time, according to the manufacturer's instructions. Briefly, after being diluted to different dilution factors based on the different cytokines, serum was incubated in the plate wells coated with capture antibody overnight at 4°C. The samples were then washed and biotin‐conjugated detection antibody was added into the plate wells for further incubation. HRP‐conjugated streptavidin was used to bind the biotin‐conjugated detection antibody to catalyze the TMB reagent. Finally, the catalytic reaction was stopped by the addition of sulfuric acid and the optical density was determined via a microplate reader (Biotek, Winooski, VT, USA, ELx800NB).

**Table 2:** Clinical data of patients and controls for ELISA

Patient	
n	20
Age (mean ± SD), year	58.85 ± 4.27
Sex	50% Male, 50% Female
Tumor location	Tunica mucosa
Lymphatic metastasis	no
Distant metastasis	no
Tumor diameter	<3 cm
Histological type	Undifferentiated
Stage	IA
Stage	I
Treatment	no
Control	
n	20
Age (mean±SD), year	60.10 ± 6.16
Sex	50% Male, 50% Female
*P*‐value (Age, patient vs control)	0.461

### 2.4 Statistical analysis

Statistical analysis was carried out using Mann‐Whitney U test with the Statistical Package for Social Science Statistics version 20 software (SPSS, IBM Corp., Armonk, NY). Differences between the two groups were considered significant if *P* values were <0.05. Data were presented as means ±SD (standard deviations). Fold change (FC) was calculated to indicate the expression tendency of cytokines in the gastric cancer group.

## 3 RESULTS

### 3.1 Differentially expressed protein analysis

**Figure 1:**
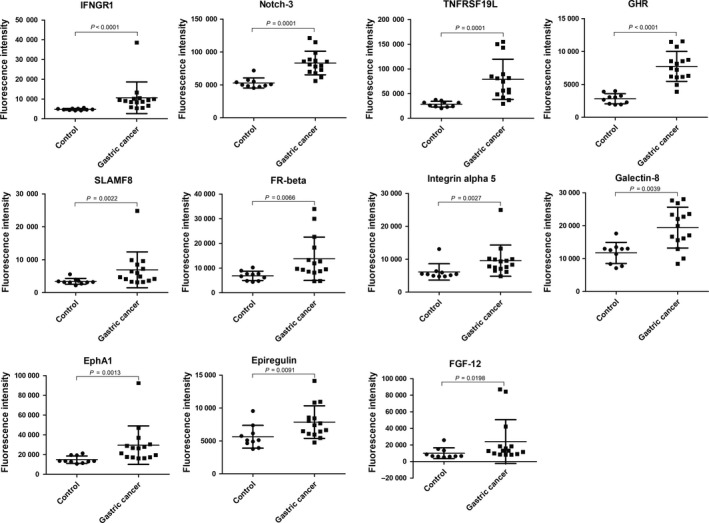
Scatter plots of differential protein levels. The levels of eleven proteins differentially expressed between gastric cancer and the control samples were detected by antibody array are shown by scatter plots with mean lines and standard deviation lines. Control group, n = 20; Gastric cancer, n = 20

**Figure 2:**
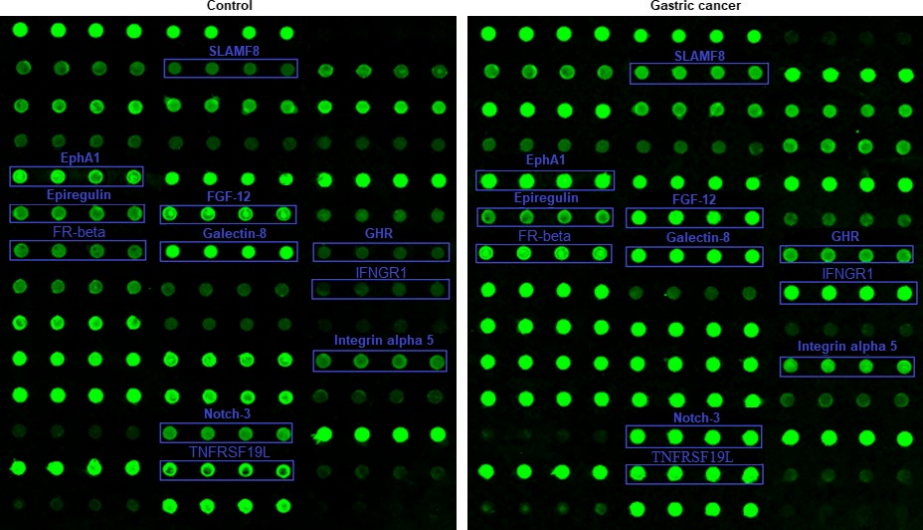
The antibody array profiles. The locations of differentially expressed proteins in the antibody array profiles are marked by blue rectangles. The levels of cytokines are shown by their fluorescence intensity which is proportional to the levels of expression

**Figure 3:**
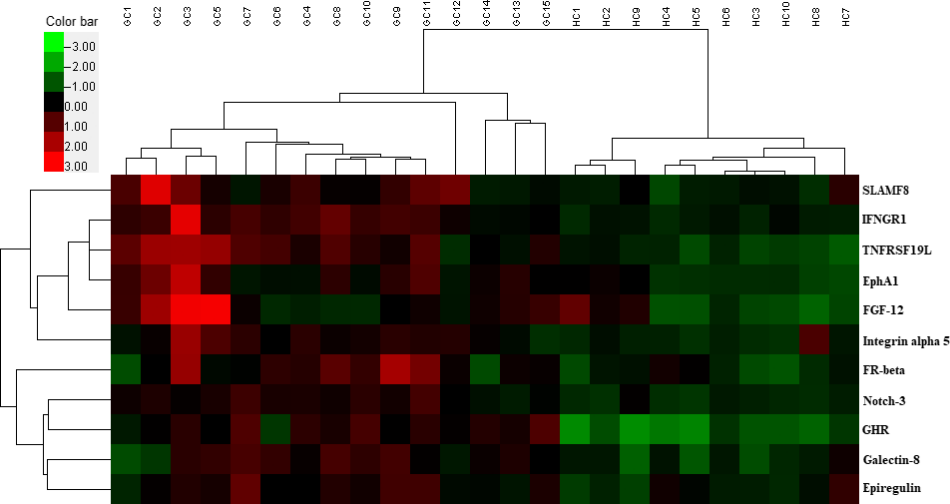
Unsupervised‐hierarchical cluster analysis. The unsupervised‐hierarchical cluster analysis accurately distinguished gastric cancer from controls, confirming differences in the expression of the eleven proteins between gastric cancer and control. Green indicates low levels of the proteins, black for median levels, and red for high levels. GC, gastric cancer; HC, healthy control

The signal values of 40 proteins were statistically analyzed by Mann‐Whitney U test to seek proteins differentially expressed between the two groups. As a result, eleven cytokines were identified that were significantly increased as compared to the control group (Table 3). The gene IDs of differentially expressed cytokines, mean signal values, *P* values and fold changes are shown in Table 3. The levels of these differentially expressed proteins are shown as a histogram using the signal values. As shown in Figure 1, the levels of the differentially expressed proteins in gastric cancer are higher than that in the healthy controls. Additionally, the representative profiles of the arrays from the two groups are shown to indicate the differences of these cytokines in gastric cancer and the controls based on the direct proportional relationship between fluorescence intensity and expression levels. As shown in Figure 2, the fluorescent signals of these proteins (blue rectangles) were clearly stronger in gastric cancer group than that in control group. Finally, an unsupervised‐hierarchical cluster was performed to distinguish the gastric cancer group and the control group using the fluorescence values of these differentially expressed proteins. The gastric cancer group and the control group were distinguished with 100% accuracy (Figure 3), further suggesting these proteins were significantly elevated in the gastric cancer group.

**Table 3:** Information about the differentially expressed cytokines

Protein name	Gene ID	GC value	HC value	Fold change (GC/HC)	*P* value (U test)
IFNGR1	3459	10 653.23	4784.44	2.227	<0.0001
Notch‐3	4854	83 220.05	52 939.13	1.572	0.0001
TNFRSF19L	84 957	78 969.55	28 658.92	2.755	0.0001
GHR	2690	7735.61	2817.25	2.746	<0.0001
SLAMF8	56 833	6933.58	3419.79	2.027	0.0022
FR‐beta	2350	13 787.23	6881.04	2.004	0.0066
Integrin alpha 5	3678	9563.02	6160.67	1.552	0. 0027
Galectin‐8	3964	19 408.19	11 720.99	1.656	0.0039
EphA1	2041	29 531.42	14 891.53	1.983	0.0013
Epiregulin	2069	7866.67	5634.43	1.396	0.0091
FGF‐12	2257	24 060.81	9952.81	2.417	0.0198

GC, Gastric cancer; HC, Heathy control.

### 3.2 ELISA validation

**Figure 4:**
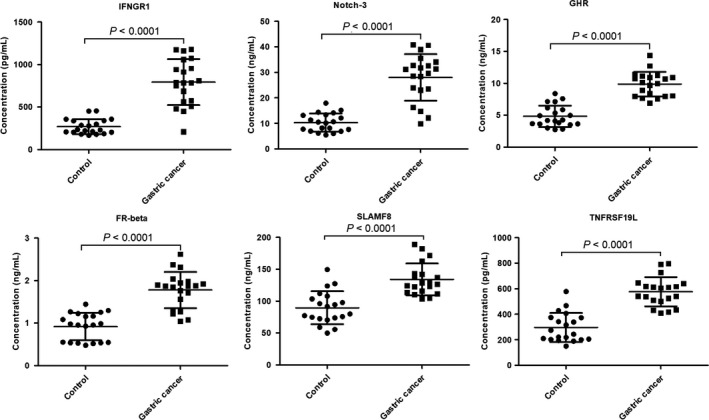
The ELISA results of six differentially expressed proteins. ELISA results of six differentially expressed proteins are shown by scatter plots with mean lines and standard deviation lines. The statistical analysis of these proteins between gastric cancer and control was performed by Mann‐Whitney U test analysis. Control group, n = 20; Gastric cancer, n = 20

Of these significantly increased cytokines, IFNGR1, Notch‐3, GHR, TNFRSF19L, FR‐beta and SLAMF8 were selected for ELISA validation due to the limits of sample volume and funding. The results of the ELISA validation are shown as a scatter plot (Figure 4), which also showed all of the six cytokines were significantly increased in patients. These results were identical to those of the microarray.

## 4 DISCUSSION

Serum is an important aspect of clinical diagnosis, as serum is readily available and procuring it is a noninvasive procedure. The ease of access to serum simplifies clinical diagnosis of diseases, provided effective biomarkers are utilized. Because of the low specificity and sensitivity of currently available serum biomarkers for gastric cancer, identification of more clinically relevant biomarkers remains an urgent need. In this study, a high‐throughput solid antibody array was utilized to screen serum biomarkers with higher clinical specificity and sensitivity and, to our knowledge, this is the first study to use antibody array techniques to identify serum biomarkers for gastric cancer.

The antibody array utilized in this study simultaneously detected 40 cytokines and identified eleven cytokines (IFNGR1, Notch‐3, TNFRSF19L, GHR, SLAMF8, FR‐beta, Integrin alpha 5, Galectin‐8, EphA1, Epiregulin and FGF‐12) significantly elevated in gastric cancer serum when compared to the controls by Mann‐Whitney U test analysis. Additionally, the unsupervised‐hierarchical cluster analysis of the microarray data accurately differentiated the gastric cancer and control groups, further detailing the differences in cytokine expression between the gastric cancer and control groups. More importantly, the significant changes of some cytokines were validated by ELISA, using fresh samples, and found to be identical to the results of the antibody array, suggesting that these cytokines increased reliably in gastric cancer serum and that these cytokines may be used as serum biomarkers of gastric cancer.

IFNGR1 is a key molecule of the IFN‐*γ* signaling pathway. Canedo et al^9^ found that IFNGR1 −56C/T gene polymorphism is associated with an increased risk of early gastric carcinoma, but there have not been any reports on the aberrant expression of IFNGR1 in gastric cancer. However, in this study, we found the levels of IFNGR1 were obviously increased in gastric cancer. Notch‐3 is a member of the Notch family of receptors and plays both oncogenic and tumor suppressor role in malignant tumors. Notch 1 and 3 genes are known to be overexpressed in intestinal and gastric carcinomas.^10^, ^11^ Likewise, we also found Notch‐3 was overexpressed in gastric cancer. TNFRSF19L is a new member of the tumor necrosis factor receptor superfamily, and is upregulated when tumor cells undergo the epithelial‐mesenchymal transition critical for cancer development.^12^ However, TNFRSF19L was found to be elevated in gastric cancer for the first time in this study. GHR, as a member of the class I cytokine receptor family, is involved in multiple biological and physiological processes contributing to cell proliferation and differentiation. GHR is a risk factor for some cancers, such as colorectal carcinoma, melanoma, uterine cervical neoplasms, breast cancer, and hepatocellular carcinoma.^13^ However, we found GHR was elevated in gastric cancer for the first time in this study, suggesting that GHR may be a risk factor for gastric cancer as well. SLAMF8, the eighth member of the SLAMF costimulatory receptors, regulates the development and function of many immune cells.^14^, ^15^, ^16^ Although Zou et al^17^ reported SLAMF8 as an independent prognosis factor in glioma and Sasaroli et al^18^ found SLAMF8 was increased in ovarian cancer, there have been few reports on the relationship of SLAMF8 and gastric cancer. In this study, we discovered that SLAMF8 was elevated in serum of gastric cancer patients. FR‐beta is the β isoform of the folate receptor. Numerous studies have shown that FRα is markedly overexpressed in ovarian, kidney, lung, brain, endometrial, colorectal, pancreatic, gastric, prostate, testicular, bladder, head and neck, breast cancers, and non‐small‐cell lung cancer.^19^, ^20^, ^21^, ^22^, ^23^, ^24^, ^25^, ^26^ However, there are few reports about FR‐beta expression in variant cancers, including gastric cancer. In this study, FR‐beta was found to be elevated in gastric cancer. Integrins are involved in key developmental processes, including cell differentiation, cell adhesion, cell migration, cell proliferation, and cell survival. It was reported that some members of the integrin alpha V family (integrin alpha V beta 5, integrin alpha V beta 6, and integrin alpha V beta 8) were up‐regulated in gastric cancer, integrin alpha 5 was a prognostic factor in early stage non‐small‐cell lung cancer, and bladder cancer cell lines expressed integrin alpha 5 at high levels.^27^, ^28^, ^29^ However, the expression of integrin alpha 5 in gastric cancer had not been evaluated before. In our study, integrin alpha 5 was shown to be overexpressed in gastric cancer. Galectin‐8 is a galectin family protein, plays an important role in endothelial cell migration, angiogenesis, cell adhesion and migration, and functions in cancer signaling pathways.^30^, ^31^, ^32^ Wu et al 33 revealed that high expression of galectin‐8 was a favorable prognostic factor for patients with gastric cancer. Similarly, this study showed Galectin‐8 was elevated in gastric cancer serum. EphA1, as the first member of the Eph receptor tyrosine kinase family, derives from erythropoietin‐producing hepatocellular carcinoma cell lines. Gastric cancer patients with overexpression of EphA1 had poorer outcomes, overall survival and relapse‐free survival than those with low levels of EphA1.^34^, ^35^ Epiregulin is a member of the epidermal growth factor (EGF) family, and is overexpressed in human gastric tumor cell lines TMK1 and MKN‐45, as are other EGF family members.^36^ FGF‐12 is a member of the FGF family, functions in the development of the central and peripheral nervous systems, connective tissue of the skeleton and the myocardia of the heart. FGF‐12 was also overexpressed in human gastric tumor cell line MKN‐45.^37^ In this study, the levels of EphA1, epiregulin, and FGF‐12 were found to be higher in gastric cancer patients than in healthy population.

In conclusion, in this study, we found eleven cytokines (IFNGR1, Notch‐3, TNFRSF19L, GHR, SLAMF8, FR‐beta, integrin alpha 5, galectin‐8, EphA1, epiregulin, and FGF‐12) that were elevated in gastric cancer serum, suggesting that these cytokines may participate in the occurrence and development of gastric cancer. Among these cytokines, Notch‐3, galectin‐8, EphA1, epiregulin, and FGF‐12 have been reported to be upregulated in gastric cancer. However, the other six cytokines, including IFNGR1, TNFRSF19L, GHR, SLAMF8, FR‐beta, and integrin alpha 5 are reported to be elevated in gastric cancer for the first time in this study, suggesting that these proteins may be novel serum biomarkers for the early diagnosis and prognosis of gastric cancer.

## 5 ETHICS APPROVAL AND CONSENT TO PARTICIPATE

All procedures using humans in this study were approved by the Ethics Committee of the Lishui Central Hospital. All participants approved participating in the study and signed written informed consent forms.

## CONFLICT OF INTEREST

The authors declare that no conflicts of interest exist.
